# Breathomics in Diabetes Management: A Noninvasive Approach for Precision Health Monitoring

**DOI:** 10.1155/jdr/7977153

**Published:** 2026-03-12

**Authors:** Nathiya Ranganathan, J. Geetha, Anbarasu Krishnan, A. S. Vickram, A. Saravanan, Jeganathan Chinnadurai, Yi-Hsun Chen, Wei-Chung Chen, I-Chen Wu, Vinoth Kumar Ponnusamy, Selvakumar Palaniappan

**Affiliations:** ^1^ Department of Microbiology, Auxilium College (Autonomous), Vellore, Tamil Nadu, India, auxiliumcollege.edu.in; ^2^ Department of Bioinformatics, Saveetha School of Engineering, Saveetha Institute of Medical and Technical Sciences (SIMATS), Chennai, Tamil Nadu, India, saveetha.com; ^3^ Department of Biotechnology, Saveetha School of Engineering, Saveetha Institute of Medical and Technical Sciences (SIMATS), Chennai, Tamil Nadu, India, saveetha.com; ^4^ PhD Program in Life Sciences, College of Life Sciences, Kaohsiung Medical University (KMU), Kaohsiung, Taiwan; ^5^ Division of Gastroenterology, Department of Internal Medicine, Kaohsiung Medical University Hospital, Kaohsiung, Taiwan, kmuh.org.tw; ^6^ Department of Medicine, Faculty of Medicine, College of Medicine, Kaohsiung Medical University, Kaohsiung, Taiwan, kmu.edu.tw; ^7^ Department of Medicinal and Applied Chemistry, Kaohsiung Medical University (KMU), Kaohsiung, Taiwan; ^8^ Research Center for Precision Environmental Medicine, Kaohsiung Medical University (KMU), Kaohsiung, Taiwan; ^9^ Department of Medical Research, Kaohsiung Medical University Hospital (KMUH), Kaohsiung, Taiwan; ^10^ Department of Food Technology, School of Applied Sciences, Papua New Guinea University of Technology, Lae, Papua New Guinea, unitech.ac.pg

**Keywords:** breathomics, diabetes management, lifestyle intervention, noninvasive diagnostics, personalized medicine, volatile organic compounds

## Abstract

The rising prevalence of diabetes necessitates accurate, noninvasive methods for early detection and personalized management. Breathomics, the investigation of exhaled volatile organic compounds (VOCs), offers a promising approach for evaluating glycemic control, oxidative stress, and metabolic alterations associated with diabetes. Specific VOCs, such as acetone, isoprene, and ethanol, reflect glucose fluctuations, lipid oxidation, and insulin sensitivity, linking metabolic changes to real‐time physiological states. Recent analytical advancements in gas chromatography–mass spectrometry, proton transfer reaction–mass spectrometry, and electronic nose technologies have enhanced the reliability and clinical applicability of breath‐based assessments. The investigations focus on integrating VOC‐based metabolic signatures with lifestyle and behavioral factors to establish a precision, noninvasive framework for diabetes management. This review also discusses key challenges that remain in standardizing breath analysis techniques, interpreting complex breath data, and implementing breath‐based technology into personalized diabetes management. Breathomics provides a novel pathway for dynamic real‐time assessment of metabolic health, bridging physiological biomarkers with lifestyle responses to enable individualized diabetes management.

## 1. Introduction

Diabetes is a metabolic disorder characterized by elevated blood glucose levels due to either diminished insulin synthesis or reduced insulin activity. Over time, this disorder can cause severe difficulties in the cardiovascular system, blood vessels, kidneys, eyes, and neurological system. Among the different types of diabetes, Type 2 diabetes (T2D) is the most common, primarily affecting adults and resulting from insulin resistance (IR) and inadequate insulin production. In contrast, Type 1 diabetes (T1D) is an autoimmune condition that leads to minimal or no insulin production [1, 2]. The global diabetes burden has grown alarmingly, affecting about 460 million people in 2019, rising to 537 million in 2021, and projected to reach 783 million by 2045. Nearly 50% of cases remain undiagnosed, and 80% occur in low‐ and middle‐income nations where poor nutrition and limited healthcare access exacerbate the crisis. The global economic cost of diabetes exceeded $966 billion in 2021 and is expected to surpass $1 trillion within the next two decades, reflecting both medical expenses and lost productivity. Despite advances in lifestyle management, medications, and insulin therapy, diabetes continues to rise, which accounts for over 2 million deaths each year, emphasizing the urgent demand for diagnostic approaches that are accessible, affordable, and noninvasive [3–6]. Conventional glucose monitoring methods, such as fasting blood glucose (FBG), postprandial glucose (PPG), oral glucose tolerance tests (OGTT), hemoglobin A1c (HbA1c), and fingerstick testing, are reliable but invasive and inconvenient for continuous use [7]. Continuous glucose monitoring improves real‐time tracking but requires implanted sensors, which remain expensive and may also cause skin irritation [8]. Moreover, HbA1c reflects only average glycemic control and cannot capture short‐term metabolic variations linked to diet or physical activity [9]. Consequently, attention has shifted toward noninvasive approaches such as salivary glucose testing, tear‐based biosensors, sweat glucose patches, urine metabolomics, and particularly breathomics—the analysis of VOCs in exhaled breath that reflects real‐time metabolic states and offers a painless, rapid, and patient‐friendly alternative for diabetes monitoring.

Breathomics, the study of volatile organic compounds (VOCs) in exhaled breath, has emerged as a promising noninvasive platform for diagnosing and monitoring diabetes. Exhaled breath is a complex biological matrix containing inorganic gases, nonvolatile compounds, and over 3500 VOCs, which are linked to metabolic processes like oxidative stress, lipid peroxidation, and ketogenesis. Key biomarkers such as acetone concentration have been reported to range from 0.3 to 0.9 ppm in healthy individuals. In comparison, elevated levels of 1.5–10 ppm are frequently observed in poorly controlled T2D patients, suggesting that acetone results from fatty acid oxidation under insulin‐deficient conditions. Isopropanol, typically measured at 0.08–0.35 ppm, has shown a dynamic response to hyperglycemia, suggesting its potential as a marker of acute glycemic fluctuations. Ethanol, pentane, and methyl nitrate are indicative of oxidative stress and glycemic fluctuations. These VOCs function collectively as metabolic fingerprints, reflecting real‐time changes in physiology and biochemistry that may aid in the early detection and monitoring of treatment and lifestyle interventions in diabetes [10–14].

Analytical platforms for detecting and quantifying VOCs vary in sensitivity, specificity, and clinical practicality. Gas chromatography–mass spectrometry (GC‐MS) remains the benchmark for VOC identification and quantification due to its high sensitivity and separation capabilities, but it is limited by lengthy analysis times and the need for specialized laboratory settings. Proton transfer reaction–mass spectrometry (PTR‐MS) and selected ion flow tube–mass spectrometry (SIFT‐MS) enable near‐real‐time vapor analysis with detection limits in the low parts per billion (ppb) range, making them suitable for clinical monitoring; however, their complexity and cost inhibit widespread adoption. Electronic nose (eNose) sensor arrays provide rapid pattern recognition of breath VOCs using cross‐reactive sensor signals but lack the chemical specificity of mass spectrometry methods and require advanced machine learning algorithms for accurate interpretation [15–17]. Despite these advances, critical challenges also remain, such as the standardization of breath collection protocols and accounting for confounding factors such as diet, physical activity, and comorbidities, which are essential to ensure reproducibility and comparability across studies. Additionally, inter‐ and intraindividual variability in VOC expression complicates biomarker validation. Furthermore, the integration of these technologies into routine clinical workflows is hampered by limited large‐scale validation studies and regulatory hurdles. This review is aimed at comprehensively evaluating the current literature on breathomics for diabetes assessment, focusing on specific VOC biomarkers, the comparative strengths and limitations of detection technologies, and the translational barriers that must be addressed to enable clinical implementation of breath‐based diagnostics [18–20].

**Figure 1:**
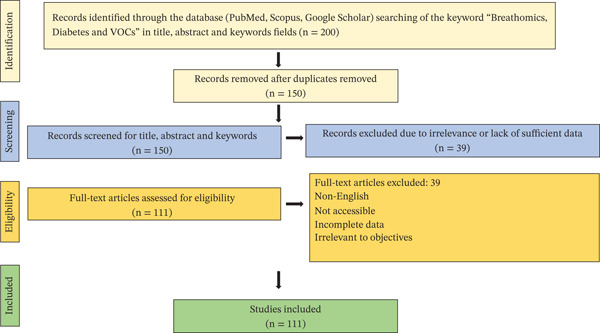
PRISMA flowchart of the literature search and study selection process.

This literature review systematically analyzed recent advances in noninvasive diabetes diagnostics and monitoring via breath analysis, focusing on VOCs and related technologies. This review is based on a comprehensive overview of 111 peer‐reviewed articles published between 2009 and 2025. The primary databases used for sourcing relevant literature included PubMed, ScienceDirect, IEEE Xplore, Google Scholar, and the World Health Organization (WHO) official website. Figure 1 shows the PRISMA flowchart of the literature search and study selection process of this review work. Relevant journals and articles were accessed through platforms and publishers such as Elsevier, The Lancet, Wiley Online Library, NCBI, MDPI, Frontiers, Springer Nature, and Academia. The key search terms include diabetes mellitus, breath biomarkers, VOCs, eNose, exercise and insulin sensitivity, probiotics and glucose metabolism, and noninvasive diagnostics. The articles were selected that focused on noninvasive diagnostic approaches, therapeutic strategies, and technological innovations related to the management of diabetes.

### 1.1. Lifestyle and Nutritional Influences on VOC Profiles in Diabetes

Lifestyle factors, such as diet and exercise, alter VOC patterns in diabetes. Low‐carb diets and fasting raise breath acetone levels, while high‐carb intake increases breath isoprene and ethanol levels. Moderate exercise elevates isoprene levels, and high‐intensity exercise increases acetone and pentane levels. These VOC shifts reflect metabolic responses in Type 1, Type 2, and prediabetic individuals.

Adults with T2D should engage in aerobic training (e.g., walking, jogging, or cycling) at moderate intensity (55%–74% HRmax; RPE 12–13) or vigorous intensity (75%–95% HRmax; RPE 14–16) frequency of at least 150 min of physical activity per week, with activity evenly distributed over a timeframe of 3–7 days and with no more than 2 consecutive rest days in between each session. Resistance training, involving all major muscle groups, should be performed on 2–3 nonconsecutive days per week, with frequency corresponding to 10–15 repetitions per set for 1–3 sets of 8–10 exercises and at a moderate or vigorous intensity. High‐intensity interval training (HIIT) utilizes short bouts of vigorous exercise (75%–95% HRmax) followed by periods of active recovery (30%–60% HRmax) and can improve both insulin sensitivity and glycemic control. It should be performed 2–3 days weekly as a part of a patient’s diabetes exercise prescription. Finally, another important aspect of exercise prescriptions for diabetes patients is to limit prolonged sedentary periods, which can be achieved by engaging in light‐intensity physical activity (e.g., standing, walking, or basic resistance exercises) every 30 min. Together, their physiological effects will promote greater cardiorespiratory fitness, lower HbA1c, reduced visceral fat, and improved insulin action and can be summarized into an evidence‐based framework to enhance metabolic outcomes for individuals with T2D. HIIT, involving bursts of 75%–95% HRmax for 10 s to 4 min interspersed with recovery, achieves greater improvements in VO_2_ max, approximately 5 mL/kg/min more than moderate continuous training, and tends to reduce HbA1c by about 0.8%. These gains in aerobic capacity and glycemic markers correspond with measurable changes in breath VOCs. Acetone levels typically increase during high‐intensity efforts due to enhanced ketogenesis, whereas isoprene levels decrease with improved metabolic efficiency. However, VOC response variability is higher during peak intensities (> 90% VO_2_max) due to altered respiratory patterns, especially in T1D [21, 22].

Consumption of dietary fiber, low‐glycemic index foods, and Mediterranean diets high in unsaturated fats and antioxidants appears to reduce oxidative stress VOC markers (aldehydes and esters) and contribute to stabilized breath acetone and isoprene levels. In contrast, ketogenic or very‐low‐carbohydrate diets tend to increase breath acetone concentrations due to increased fat oxidation and ketone body production. Studies with intervention lengths of 4–12 weeks have shown substantial shifts of VOC markers, which correlate with concurrent improvements in fasting glucose and HbA1c. VOC profiling is most sensitive in early‐stage T2D and prediabetes, where breath biomarkers demonstrate larger effect sizes compared to advanced or medicated disease states that show greater metabolic instability. Additionally, glycemic variability, physical fitness levels, and medication use (e.g., metformin and insulin) modulate VOC expression, necessitating personalized baselines and longitudinal monitoring for accurate interpretation [21–23].

## 2. Biological Basis

VOCs are characterized by their small size, carbon‐based structure, and tendency to readily evaporate into a gas at typical room temperatures, which can be detected in human breath, urine, perspiration, and other physiological fluids [18, 19]. Endogenous metabolic processes or interactions with environmental variables derive these chemicals. VOCs are currently utilized extensively as noninvasive biomarkers for various physiological and pathological states, including metabolic disorders such as diabetes. Diabetes dysregulates glucose metabolism, induces oxidative stress, and activates ketogenesis, leading to the formation of diverse organic volatiles. These evaporative organic compounds can serve as real‐time indicators of metabolic changes and illness progression, especially when traditional diagnostic techniques are invasive or limited in their ability to provide dynamic information [20]. In diabetes, metabolic processes are associated with glucose metabolism, oxidative stress, and ketogenesis, leading to the production of a variety of VOCs. Diabetes may lead to elevated levels of reactive oxygen species (ROS), which in turn initiate oxidative stress and affect metabolic function. Diabetes is known to change fatty acid metabolism and ketogenesis, leading to the production of organic volatiles [24]. The role of VOCs in diabetes and metabolism is outlined in Table 1.

**Table 1:** Role of volatile organic compounds (VOCs) in diabetes and related metabolic processes.

Metabolic process	Key VOCs identified	Concentration and correlation	Underlying mechanism	Clinical relevance	Comparison insights	References
Glucose metabolism	Acetone (0.74–2.92 ppm), isoprene, ethanol, methanol, and aldehydes	Breath acetone ranges ~0.5–3 ppmv and correlates positively with blood glucose and HbA1c in T1DM (*R* = 0.75 − 0.88). Isoprene has variable correlation (*R* = 0.4 − 0.7). Ethanol/methanol levels vary with gut microbiota and metabolism.	Altered glycolysis and the TCA cycle shift metabolite fluxes, producing characteristic VOCs such as acetone and isoprene.	Noninvasive glucose monitoring potential. Breath VOCs reflect metabolic rate and glucose control. Acetone is a consistent hyperglycemia marker, especially in T1DM.	Acetone shows strong predictive power for hyperglycemia across multiple studies. Isoprene and alcohols provide complementary or confounding signals. Microbial contributions complicate ethanol/methanol as biomarkers.	[25, 26]
Oxidative stress	Aldehydes (malondialdehyde and hexanal), ketones, alcohols, and esters	Elevated aldehydes up to 0.3 ppmv were noted in diabetes. Aldehydes correlate with oxidative stress markers. Ketones and esters show variable presence linked to lipid peroxidation and microbiota activity.	High glucose increases ROS production, causing lipid peroxidation, releasing aldehydes and other VOCs indicative of oxidative damage.	Indicates oxidative damage contributing to diabetic complications and worsening insulin sensitivity; potential early biomarker of disease progression.	Aldehydes correlate better with oxidative stress severity than ketones. Esters’ origin is uncertain, but gut microbiota is implicated. VOC patterns differ by diabetes type and duration.	[27, 28]
Ketogenesis	Acetone (up to ~3 ppmv), acetoacetate, *β*‐hydroxybutyrate, isopropanol, and ethanol	Acetone up to ~3 ppm in ketosis; up to 1250 ppm in ketoacidosis; strong correlation with blood *β*‐hydroxybutyrate (*R* = 0.82 − 0.89).	Fatty acid breakdown in insulin deficiency produces ketone bodies, which are exhaled as VOCs.	Breath acetone robustly monitors ketosis and diabetic ketoacidosis noninvasively. Isopropanol and ethanol provide additional diagnostic value.	Acetone is the most reliable marker of ketosis. Other VOCs, like isopropanol, assist in differentiating metabolic states.	[29]

### 2.1. Glucose Metabolism

Glucose metabolism refers to the process by which glucose (a simple sugar) is metabolized into energy in the form of ATP. This process supports biological functions and is regulated by hormones such as insulin and glucagon. Glucose metabolism refers to the process by which glucose (a simple sugar) is metabolized into energy in the form of ATP [30]. The ability of insulin to regulate glucose metabolism is primarily mediated by two pathways: insulin‐mediated glucose uptake (IMGU), which affects glucose uptake by cells, and glucose‐stimulated insulin secretion (GSIS), the process by which the pancreas secretes insulin in response to elevated blood glucose levels. Specifically, insulin facilitates metabolic requirements by initiating the IMGU pathway, promoting increased glucose uptake by skeletal muscle and adipose tissue while simultaneously suppressing hepatic glucose production. The effect of insulin is biochemically mediated by its binding to the *α*‐subunit of the insulin receptor, thereby stimulating intracellular signaling through the insulin signaling pathway. This initially involves a conformational change in the receptor complex, which then leads to the receptor’s beta subunit being autophosphorylated. After the autophosphorylation of the beta subunit and phosphorylation of the insulin receptor substrates (IRSs) by kinases, phosphatidylinositol 3‐kinase (PI3K) becomes activated. Following several upstream signaling events, the final effector pathway results in the translocation of the GLUT4 transporter from intracellular stores to the plasma membrane of skeletal muscle cells, thereby increasing cellular glucose uptake. GLUT4 transporters are stored within intracellular vesicles, and their movement to the surface of the cell (exocytosis) is accelerated by both insulin and exercise. Insulin specifically enhances GLUT4 translocation to the cell surface of skeletal muscle cells, thereby enabling glucose to enter these cells and be converted into glycogen for storage [31, 32].

Glucose metabolism, the conversion of simple sugar (glucose) into energy, is integral to multifaceted health; impairment of glucose metabolism can lead to diseases, with diabetes being the most prominent example. Understanding glucose metabolism is crucial for effectively managing diabetes. Organic volatiles are associated with metabolic activities, such as glucose metabolism. The identification of these organic volatile biomarkers (VOCs) in breath and bodily fluids offers a noninvasive approach to assessing glucose levels and facilitating the diagnosis and treatment of diabetes [25, 33].

Insulin and glucagon are hormones that regulate glucose metabolism, the primary source of energy for humans. However, diabetes disrupts this process, resulting in elevated blood glucose levels. Recent studies suggest that organic volatiles released by humans, which serve as indicators of metabolic activity, could serve as markers. Breath analysis suggests that detecting acetone and isoprene may correlate with the degree of blood sugar control. This would be a promising technique for the timely detection and control of diabetes. Glucose metabolism results in the production of several VOCs, including a range of volatile alcohols and ketones, during glycolysis and the tricarboxylic acid (TCA) cycle, which are byproducts of glucose metabolism. In cases of hyperglycemia, advanced glycation end products (AGEs) may be produced. AGEs interact with proteins or lipids, causing lipid peroxidation and the generation of ROS by electron transfer. Through this process, organic volatiles (VOCs) are released [34].

**Figure 2:**
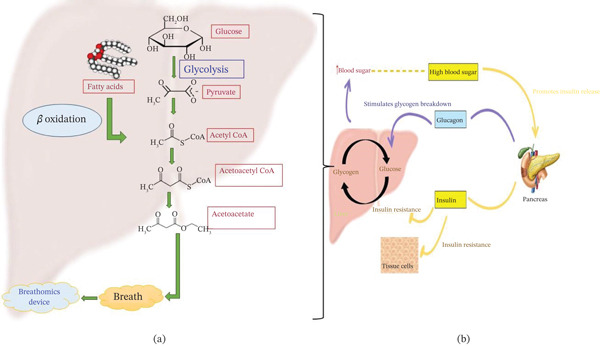
The image illustrates the correlation between glucose regulation and lipid metabolism in diabetes. (a) Insulin resistance or deficiency impairs glucose utilization, leading the (b) liver to increase fatty acid oxidation and ketone body formation. The resulting formation of VOCs released through the breath serves as a noninvasive biomarker for diabetes monitoring.

Figure 2 illustrates the metabolic pathway of glucose metabolism. Cellular oxidative stress, a cellular condition caused by the generation of free oxygen radicals (ROS) that surpass the body’s antioxidant defenses, has been implicated in diabetes. Even though oxygen radicals naturally arise from cellular metabolic processes, an excess concentration can damage cells if not properly neutralized, contributing to diabetic complications [35]. Diabetes causes oxidative stress through multiple methods, including the buildup of glycolytic intermediates, increased activity of the polyol pathway, the generation of AGEs, stimulation of Sphingosine‐Dependent Protein Kinase 1 (SDK1) or Protein Kinase C (PKC), and upregulation of the hexosamine biosynthetic pathway (HBP) [36, 37]. The disruption of balance triggers a cascade of reactions that have detrimental consequences for the body. Overproduction of oxygen radicals (ROS) can have a detrimental effect on vital cellular constituents, including polypeptides, lipids, triglycerides, and genetic materials (DNA), resulting in reduced cellular function and altered physiological processes. Oxidative stress can cause inflammation and disrupt biological systems, resulting in the appearance of health issues, such as metabolic disorders, including diabetes [38]. According to Caturano et al., oxidative imbalance plays a key role in the development of diabetes mellitus, leading to the formation of various biomarkers. Among these are malondialdehyde and 4‐hydroxynonenal as indicators of lipid peroxidation; 8‐oxo‐2’‐deoxyguanosine and high nitrite levels as indicators of DNA oxidation; oxidative damage to protein like carbonyl compounds or AGEs such as glycated hemoglobin; lower levels of antioxidants like glutathione or vitamins A, C, and E; and diminished activity in enzyme antioxidants like superoxide dismutase or catalase, all of which are evidence of an aggravated oxidative state. These oxidative stress biomarkers are currently being assessed using noninvasive “breathomics” approaches to monitor diabetes [39–41].

**Figure 3:**
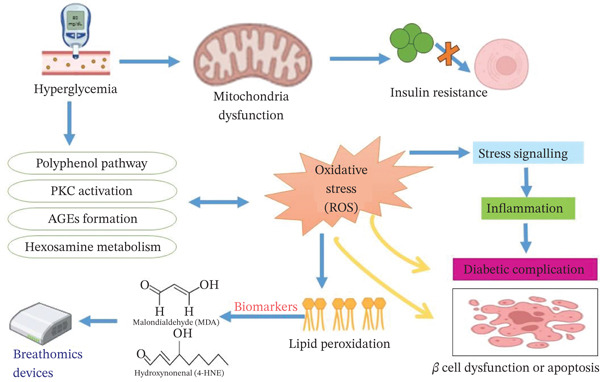
The mechanism linking hyperglycemia‐induced oxidative stress to diabetic complications and potential detection via breathomics. PKC, Protein Kinase C; AGEs, advanced glycation end products.

The condition of oxidative imbalance contributes significantly to the initiation and progression of the diabetic state and its subsequent complications, including heart and blood vessel problems. In diabetes, an excess of free radicals (ROS) damages cells and tissues, leading to IR, inflammation, and long‐term complications, such as retinopathy and neuropathy. Organic volatiles (VOCs) have been linked to diabetes and related disorders, but the interaction between VOC exposure and oxidative stress in diabetes is complex and needs further investigation. Oxidative stress can influence insulin signaling, cause IR, and accelerate the progression of diabetes. It also contributes to many diabetic challenges like cardiovascular disease, retinal damage, nerve damage, and kidney damage. Excess oxygen‐free radicals can either directly produce VOCs or impact metabolic pathways, releasing chemicals such as alcohols, ketones, and esters [27, 28]. The mechanism linking hyperglycemia‐induced oxidative stress to diabetic complications and potential detection via breathomics is given in Figure 3.

### 2.2. Ketogenesis

In diabetes, insufficient insulin production often leads to increased ketogenesis, the production of ketone bodies, resulting in elevated levels of acetone and other organic volatile substances in our breath. VOCs, especially acetone, could act as nonintrusive biomarkers for the assessment and surveillance of diabetes, particularly in detecting diabetic ketoacidosis (DKA). In individuals with diabetes, especially those with T1D, low insulin levels cause the hepatic cells to produce excessive ketone bodies, including acetoacetate, *β*‐hydroxybutyrate, and acetone. As glucose is not efficiently used for energy, the human body relies on fat as its primary fuel source. This elevated ketogenesis leads to increased levels of exhaled breath organic volatiles like acetone, making breath analysis a valuable nonintrusive instrument for monitoring ketone levels, particularly in diagnosing DKA, characterized by a severe, elevated blood sugar level, elevated ketones, and metabolic acidosis, where acetone exhaled in the breath contributes to the fruity odor commonly associated with the condition. Other VOCs, such as isopropanol and ethanol, have also been linked to diabetes, and studies suggest that isoprene may be related to cholesterol synthesis in diabetic patients. Overall, breath analysis for these VOCs offers a promising method for monitoring and potentially diagnosing diabetes [20, 25].

### 2.3. VOC Mechanisms and Clinical Implications in Diabetes

Exhaled breath VOCs are real‐time measures of the biochemical changes that are occurring in diabetes mellitus, which are alterations in glucose metabolism, fatty acid oxidation, ketogenesis, and oxidative stress. The mechanism is through insulin deficiency or resistance, shifting hepatic acetyl‐CoA away from the TCA cycle toward ketone bodies, producing acetoacetate and *β*‐hydroxybutyrate, which spontaneously decarboxylate to acetone, a VOC and biomarker of altered energy metabolism. Several studies have identified a distinct VOC profile in the exhaled breath of diabetic patients compared to healthy individuals, revealing significant quantitative and qualitative differences linked to altered glucose metabolism and oxidative stress. Among these, acetone remains the most consistent and elevated biomarker across studies. Using methods such as gas chromatography, sensor array technology, and solid‐phase microextraction (SPME) coupled with GC‐MS, acetone levels in healthy subjects ranged from 0.044 to 2.744 ppm, whereas in diabetic patients, they increased markedly, ranging from 2.2 to 21.0 ppm in T1D and 1.76 to 9.4 ppm in T2D. This reflects a 10–50‐fold elevation above nondiabetic levels, confirming acetone’s role as a metabolic surrogate for ketogenesis and insulin deficiency. Other VOCs, such as ethylbenzene, xylene, toluene, ethane, pentane, and propane, were detected at trace concentrations (0.0002–0.04 ppm), likely originating from lipid peroxidation and the oxidative degradation of unsaturated fatty acids, supporting their mechanistic association with oxidative stress in diabetes. Isoprene levels varied inconsistently, with some studies reporting reductions in poorly controlled T2D, potentially reflecting altered cholesterol synthesis pathways. In contrast, alcohols such as ethanol, methanol, and isopropanol showed modest but significant increases of 0.029 ppm in diabetic breath compared to controls, possibly linked to hepatic metabolic shifts and enzymatic conversion from acetone. Additionally, higher alkanes, such as tridecane, undecane, and trimethylhexane derivatives, were detected in Type 2 diabetics but not in healthy individuals, indicating dysregulation of complex lipid metabolism. Collectively, these findings suggest that diabetes induces a characteristic VOC fingerprint dominated by elevated acetone and secondary alcohols, with supporting changes in hydrocarbons and aldehydes reflecting oxidative and metabolic imbalance. When analyzed by GC‐MS or sensor arrays, VOC patterns can differentiate diabetic states with reported accuracies exceeding 80%–90%, offering a noninvasive diagnostic and monitoring platform superior to single‐analyte blood testing in terms of real‐time metabolic reflection. Future directions emphasize the integration of multi‐VOC panels with metabolic flux and lifestyle data to enhance sensitivity and specificity, paving the way for personalized breath‐based diabetes monitoring [42–47].

#### 2.3.1. Acetone as a Noninvasive Breath Biomarker for Diabetes

Acetone, an organic volatile molecule exhaled through the breath, is a reliable and nonintrusive indicator for monitoring T1D mellitus. It is produced during ketone metabolism by the oxidative degradation of nonesterified saturated fatty acids, followed by the spontaneous or enzymatic decarboxylation of acetoacetate. The amount of acetone detected in breath closely matches the concentration of ketones in the bloodstream, including *β*‐hydroxybutyrate, and tends to increase in diabetic conditions. In healthy persons, breath acetone levels are usually below 0.8 ppm, whereas diabetic patients often exceed 1.8 ppm. In severe cases like DKA, concentrations may rise dramatically to as high as 1250 ppm. Elevated levels are also observed during prolonged fasting or when following a ketogenic diet. Thus, measuring breath acetone provides a better, nonintrusive way to evaluate diabetic status and metabolic health than blood glucose testing alone [48–50].

Breathomics is a novel, noninvasive diagnostic method for studying metabolic alteration within diabetes. The VOCs, including acetone, isopropanol, ethanol, and carbonyl, indicate changes in glucose metabolism, fatty acid oxidation, and oxidative stress. The concentration of the VOCs differs markedly among healthy individuals, diabetic persons, and those with ketoacidosis (DKA). Comparison of the VOC profile among groups provides insight into the progression of the disease and the utility of breath composition in detecting blood glucose levels (Table 2).

**Table 2:** Comparison of key breath VOC biomarkers in healthy, diabetic, and DKA patients.

VOC biomarker	Healthy individuals	Diabetic patients	DKA	Features	References
Acetone	< 0.8 ppm (or 0.3–0.9 ppm)	> 1.8 ppm	Up to 1250 ppm (commonly 75–1250 ppm)	End product of fatty acid oxidation; reflects ketone metabolism and glycemic control	[13, 51]
Beta‐hydroxybutyrate	< 0.4 mmol/L (fasting)	< 0.6 mmol up to 1250 ppm (commonly 75–1250 ppm) l/L	0.6–3.0 mmol/L (at risk); > 3.0 mmol/L, often 5–10 mmol/L or higher (diabetic ketoacidosis [DKA])	Principal ketone body produced during fatty acid oxidation; serves as a major indicator of ketogenesis and correlates strongly with breath acetone levels in diabetes and DKA	[52, 53]
Isopropanol	0.006–0.062 ppm	0.008–0.782 ppm	Can reach very high levels; often discussed in terms of blood concentrations. Blood isopropanol levels in DKA can average 15.1 mg/dL, with a range up to 170 mg/dL, without exposure to external isopropanol	Secondary alcohol from acetone reduction (↑ NADH/NAD^+^ ratio)	[54]
Isoprene	0.08–0.30 ppmv	0.08–0.30 ppmv	Not a primary DKA biomarker	Product of cholesterol biosynthesis; correlates with oxidative stress	[55]
Ethanol	< 0.05	0.1–3 ppm	Usually low unless exogenous intake	Endogenous microbial fermentation & altered hepatic metabolism	[46]

## 3. Volatile Associated With Glycemic Status and Its Clinical Relevance

### 3.1. Key VOCs in Diabetes

The occurrence of organic volatiles in exhaled air is helpful for both early diagnosis and real‐time assessment of diabetes. Volatile compounds (e.g., acetone, isoprene, ethanol, and isopropanol) are associated with metabolic changes in patients with diabetes. These exhaled breath compounds can be identified using methods that employ both gas chromatography and mass spectrometry (Table 3).

**Table 3:** Key VOCs in diabetes.

Biomarker	Significance	Mechanism	Measurement and breath concentration range	Comparative insights	Clinical applications	References
Acetone	Elevated breath acetone indicates diabetes, especially in diabetic ketoacidosis (DKA)	Formed from fatty acid *β*‐oxidation during insulin deficiency	Greater than 1.8 ppm in diabetics; correlates with blood ketones and glucose; sharp rise in DKA	Most extensively validated VOC biomarker; high sensitivity and specificity; robust indicator of ketosis and metabolic control	Noninvasive monitoring of ketosis and DKA; potential surrogate for blood ketone levels; useful in breath‐based diabetes diagnostics	[12]
Isopropanol	Emerging marker linked to glycemic control and hypoglycemia	Produced enzymatically from acetone by alcohol dehydrogenase	0.008–0.078 ppm in diabetic breath; correlates with acetone and glucose	Promising complementary biomarker with moderate diagnostic accuracy; useful for hypoglycemia detection; less studied but growing interest	Real‐time monitoring of glycemic variability and hypoglycemia; potential wearable sensor target	[48]
Ethanol	Elevated in diabetic breath, possibly due to metabolic changes or gut microbiota	May arise from altered metabolism and gut microbial activity	Breath ethanol levels vary widely; less consistent correlation with glucose or ketosis	Less specific and sensitive than acetone or isopropanol; influenced by external factors; useful as a complementary biomarker but limited standalone diagnostic value	Complement to acetone/isopropanol in multibiomarker panels; insight into microbiota interactions and metabolic dysregulation	[48]
Isoprene	Indicative of lipid metabolism and cholesterol synthesis alterations in diabetes	Byproduct of cholesterol biosynthesis and lipolysis	0.05–0.3 ppm; variable correlation with metabolic parameters	Less reliable as a direct glycemic marker; valuable for lipid metabolism and cardiovascular risk assessment in diabetes	Useful for metabolic phenotyping; monitoring lipid‐related complications (e.g., cardiovascular disease)	[47]

### 3.2. Breath Signatures in Different Stages of Diabetes

The changing composition of our breath, particularly in exhaled air, can reflect different stages of diabetes. For example, in T1D, a significant rise in the level of acetone, particularly when the person is in the dangerous state of DKA, is the critical warning sign. This makes monitoring breath a valuable, nonintrusive approach for detecting ketoacidosis in its early stages. T2D exhibits specific and substantial changes in organic volatiles, including increased concentrations of isoprene and alkanes, which indicate free radical oxidative stress, as well as alterations in ethanol and aldehydes. In prediabetes individuals, minor elevations of VOCs like acetone and isoprene may indicate preclinical early warning signs that an individual is beginning to lose blood sugar balance [56, 57].

#### 3.2.1. T1D

A noninvasive method that may prove helpful for T1D management is breath analysis. Acetone, a ketone body, is among the most frequently observed indicators. Acetone levels can rise due to increased fatty acid oxidation when insulin is deficient. In T1D, DKA triggers lipid metabolism for energy, leading to elevated acetone production that is expelled through the breath, making breath acetone a noninvasive, rapid, and early diagnostic indicator of DKA. In addition to acetone, isoprene is another VOC that has been identified at higher levels in the breath of individuals with T1D. Isoprene is produced from a precursor formed via the mevalonate pathway (also responsible for cholesterol biosynthesis), and elevated concentrations may indicate alterations in lipid metabolism and oxidative stress, both common in T1D. Several VOCs, including carbon monoxide (CO), methyl nitrate, and dimethyl sulfide, are elevated in the breath of individuals with T1D, whereas CO in T1D results from heme degradation and increased free radical production.

Methyl nitrate and dimethyl sulfide arise due to altered metabolic activities in T1D. These organic volatiles reflect a biochemical shift associated with T1D. The identification of these exhaled breath components paves the way for a growing interest in breath omics as a valuable diagnostic instrument in diabetes research [58–62]. Recent investigations have demonstrated the potential of VOC analysis in breath and urine for detecting diabetes. A preliminary clinical study involving a home‐based breath collection device for T1D patients demonstrated that artificial intelligence algorithms analyzing VOC profiles could predict blood glucose levels with over 80% accuracy. This highlights the significant potential of breath organic volatiles for minimally invasive glucose monitoring [63].

#### 3.2.2. T2D

In patients with T2D, analysis of exhaled breath has emerged as a prominent noninvasive technique for screening metabolic changes through VOCs. Although acetone is detectable in the breath of T2D patients, its concentrations are typically lower and more variable than those observed in T1D. This inconsistency arises from residual insulin function in T2D, which helps limit ketone production. Consequently, the relationship between breath acetone levels and blood ketones is less reliable in T2D than in T1D; however, increased breath acetone can still be detected when blood sugar control is poor, during prolonged fasting, or with increased IR [64].

T2D patients were exhaling a mix of VOCs that signal metabolic dysregulation in addition to acetone. Specific molecules, such as isopropanol, ethylene, ammonia, toluene, and several hydrocarbons (pentane and hexane), were detected at higher levels in the breath of patients with T2D. Each of these VOCs relates to specific disease processes, such as oxidative stress, protein hydrolysis, and lipid peroxidation. For example, the presence of abundant ammonia could indicate altered nitrogen metabolism and kidney involvement, while isopropanol may be a metabolite of acetone reductive metabolism. Ethylene and toluene may indicate oxidative degradation of lipids, as well as incidentally altered metabolic processes in the presence of reductive processes triggered by external or internal factors. Similar patterns and the intensity of this specific mix of respiratory biomarkers are a noninvasive method for identifying and monitoring T2D [56, 65].

#### 3.2.3. Prediabetes

Prediabetes is a condition in which the body becomes less sensitive to insulin, accompanied by slightly elevated blood glucose levels. There appears to be an association between prediabetes and early changes in exhaled VOCs. Small changes in VOCs, such as acetone and isoprene, are biologically relevant in detecting metabolic abnormalities and oxidative stress. The detection of these breath biomarkers could represent a promising, noninvasive method for initial prediabetes detection and, therefore, an opportunity to prevent treatment through lifestyle changes or medical interventions [66, 67].

## 4. Lifestyle Influence

Lifestyle behaviors significantly influence metabolic processes and the VOCs in exhaled breath, which serve as biomarkers. For individuals with or at risk for metabolic diseases, including diabetes, lifestyle modifications such as diet and food intake, physical activity intensity, and nutritional supplements have a profound impact on metabolic processes, including lipid oxidation, glucose metabolism, and inflammation. Shifts in metabolism are reflected in breath volatile organic molecules, including acetone, isoprene, nitric oxide (NO), and hydrocarbons, making breathomics a valuable tool for monitoring metabolic changes associated with these behaviors. Breath analysis is also noninvasive and facilitates improved compliance, as well as real‐time monitoring of the effects of behaviors on disease progression or response to therapy. Integrating lifestyle assessment with breath‐based diagnostics may thus provide a powerful platform for personalized, preventive, and noninvasive management of diabetes.

### 4.1. Dietary Patterns

Diabetes management relies heavily on diet, which has a direct impact on exhaled biomarkers.

### 4.2. Low Carbohydrate and Ketogenic Diets

**Figure 4:**
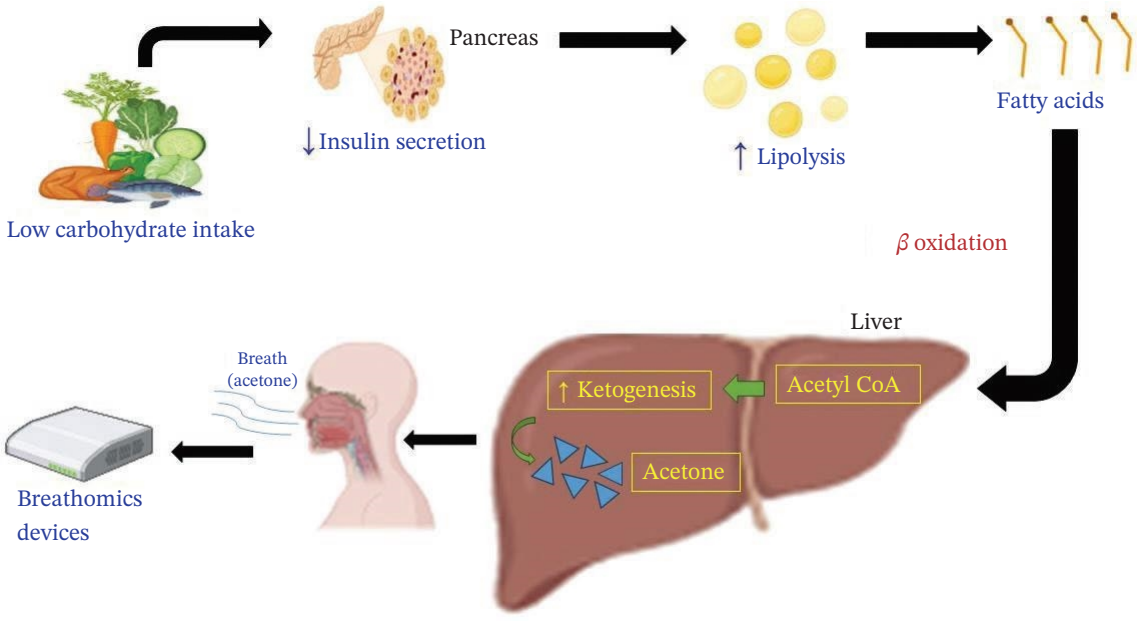
Metabolic pathways illustrating ketogenesis from low carbohydrate intake, leading to breath acetone production and detectable by breathomics.

Ketosis is the result of a nutritional approach characterized by low carbohydrate intake, high fat intake, and moderate protein intake. In this situation, the body uses fat as its primary energy source rather than carbohydrates. The liver synthesizes ketone substances, including acetone, which then enter the bloodstream and are exhaled. In individuals with diabetes, particularly those with poor glycemic control or IR, the presence of acetone in the breath may indicate enhanced fat metabolism and potential disturbances in glucose regulation. Monitoring acetone levels through breathomics provides real‐time insights into how well these dietary strategies improve insulin sensitivity and glucose metabolism in diabetic patients. In persons with T2D and prediabetes, ketogenic and low‐carbohydrate diets significantly elevate breath acetone levels, reflecting enhanced lipid oxidation and diet‐induced metabolic shifts. Breath acetone shows strong correlations with blood acetoacetate (*r* = 0.89) and *β*‐hydroxybutyrate (*r* = 0.82), validating its reliability for tracking nutritional ketosis. Portable nanotechnology‐based breath sensors enable real‐time, noninvasive monitoring and outperform urine tests for early ketosis detection. Compared with blood or urine‐based measurements, VOCs provide earlier, dynamic insights into fat metabolism and behavioral responses to diet, exercise, and supplements, improving patient engagement and reducing hospitalization risks. Methodological advances in nanosensors continue to enhance the sensitivity, cost‐effectiveness, and applicability of breathomics in lifestyle‐based diabetes management (Figure 4) [68–71].

### 4.3. High‐Fiber Diets and Diabetes

A fiber‐rich, plant‐based diet, combined with complex carbohydrates, is likely to provide the best results for glycemic control. It slows carbohydrate absorption, improves insulin sensitivity, and changes the gut microbiome. Gut bacteria can ferment dietary fiber and produce short‐chain fatty acids (SCFAs), such as acetate, propionate, and butyrate, which are essential for maintaining metabolic health. SCFAs can also modulate the production of exhaled organic volatiles (VOCs). In the diabetes population, it has been suggested that high‐fiber diets limit the levels of harmful breath organic volatiles, such as acetone and ethane, that are linked to inflammation and free radical damage from oxidative stress. Additionally, it can increase the production of organic volatiles, which is related to improved metabolic function. Thus, breathomics can provide valuable insights into how high‐fiber diets affect blood glucose levels and overall metabolic health in individuals with diabetes [72, 73]. High‐fiber diets, containing both soluble and insoluble fibers, effectively lower HbA1c by 0.3%–1.0%, improve fasting and PPG control, and enhance insulin sensitivity in T2D and prediabetes. VOCs show promise as noninvasive biomarkers for real‐time monitoring of glucose metabolism, with overall predictive accuracy of 82%–85%. In comparison to standard blood glucose and HbA1c measures, VOCs reflect the more dynamic, repetitive metabolic responses to diet and lifestyle interventions, such as high‐fiber feeds, food habits, and health behaviors. Therefore, a high‐fiber feeding paradigm integrated with VOC monitoring could improve individualized and personalized diabetes self‐management by supporting ongoing tracking of health, metabolic health, adherence, and changes toward improved health [73, 74].

### 4.4. Antioxidant‐Rich Diets and Diabetes

Antioxidant‐rich diets, including vegetables, fruits, and whole‐grain products, may help alleviate oxidative imbalance, which is often seen in diabetic conditions and can trigger an inflammatory response and harm the lining of blood vessels and tissues. In people with diabetes, oxidative damage can be measured by the amount of harmful byproducts, ethane and pentane, produced by lipid peroxidation. These markers can be observed in exhaled breath, offering an indication of the extent of oxidative damage [75, 76]. Randomized controlled trials report that antioxidant supplements, such as vitamins C and E and polyphenols, significantly reduce oxidative stress markers, such as malondialdehyde, by 16%–25%, and increase key antioxidant enzymes, such as superoxide dismutase, by about 18%, correlating with decreased fasting glucose and HbA1c levels in T2D and prediabetes subjects. These changes are paralleled by improved vascular inflammation markers and insulin sensitivity. VOC measurements have demonstrated 82%–85% predictive accuracy for glycemic changes and oxidative stress status, providing a dynamic monitoring advantage over traditional static biomarkers like fasting glucose or HbA1c. This allows real‐time assessment of metabolic responses to antioxidant‐rich diets and lifestyle changes, enhancing personalized diabetes management [77, 78].

### 4.5. Physical Activity

Physical activity is extremely important for diabetes management, as it enhances insulin action and improves glucose regulation. Physical activity enhances skeletal muscle insulin sensitivity and increases fatty acid oxidation. Regular physical activity confers health benefits for both the management and prevention of T2D. Furthermore, it offers a wide range of health benefits to individuals with both T1D and T2D [79].

Physical activity enhances the absorption of blood sugar by contracting muscles. The regulation of blood glucose during exercise involves hepatic glucose production through both glycogenolysis and gluconeogenesis, as well as the mobilization of unbound fatty acids and partially amino acids. The level and duration of physical activity are key determinants of glucose metabolism. Initially, muscle glycogen, a branching polymer of glucose, serves as the predominant energy source. As activity continues and glycogen supplies diminish, muscles rely on circulating blood sugar and free fatty acids from adipose tissue. At this point, hepatic glucose output shifts predominantly from glycogenolysis toward gluconeogenesis. Glucose uptake in muscles occurs through two pathways: insulin‐dependent at rest and postprandially and non‐insulin‐dependent during exercise, which aids glucose transport into contracting muscles alongside glycogen breakdown. GLUT4 is the primary insulin‐dependent transporter; however, in T2D, its function is impaired. Both aerobic and resistance exercise increase GLUT4 levels, improving glucose uptake despite IR. During moderate‐intensity exercise, muscle glucose uptake exceeds hepatic glucose production, leading to a decrease in blood glucose levels. Exercise reduces plasma insulin levels, decreasing the risk of hypoglycemia unless exogenous insulin is used. Exercise also increases insulin‐dependent and non‐insulin‐dependent glucose uptake in skeletal muscle, primarily by upregulating GLUT4 transporters, which are impaired in T2D. Both aerobic and resistance training improve glycemic control, reduce fasting glucose and HbA1c levels (by approximately 0.3%–0.6%), and enhance fatty acid oxidation, contributing to better overall metabolic health. Regular physical activity also benefits vascular health by increasing NO production and improving mitochondrial function and metabolic flexibility, as shown by changes in VOCs such as acetone, isoprene, and ethanol in breath analyses [80–84].

Clinical trials show that structured physical activity can significantly enhance aerobic fitness, muscle strength, and flexibility, with improvements in body composition, blood pressure, and lipid profiles. According to American Diabetes Association guidelines, ≥ 150 min/week of moderate‐intensity aerobic exercise combined with resistance training demonstrates meaningful reductions in HbA1c (about 0.3%–0.4%) alongside improved quality of life and reduced diabetes‐related complications. However, adherence remains a critical factor for sustained benefits, with evidence suggesting that thrice‐weekly supervised sessions yield better glycemic outcomes than less frequent exercise. Importantly, exercise‐induced metabolic improvements can be monitored noninvasively using breath VOC profiling. Postexercise reductions in breath acetone reflect decreased reliance on fat metabolism due to improved glucose uptake, elevated NO levels indicate enhanced endothelial function, and alterations in VOCs, such as isoprene and ethanol, correspond to better mitochondrial efficiency and metabolic flexibility. These breathomics biomarkers provide real‐time, sensitive measures of the physiological benefits of physical activity in individuals with diabetes, supplementing traditional blood‐based metrics [63, 85–87].

### 4.6. Supplementation Modulates Exhaled Biomarkers

Nutritional supplementation can significantly influence metabolic processes and corresponding breath biomarkers in individuals with diabetes. Antioxidant supplements, such as vitamin C and vitamin E, have been shown to reduce oxidative stress, resulting in decreased exhaled markers, including ethane and pentane. Omega‐3 fatty acids reduce inflammation and improve cardiovascular function, endothelial function, and NO levels. Additionally, probiotics/prebiotics enhance gut microbiota, impacting systemic metabolism and reducing IR; daily supplementation trials report ~0.4‐unit improvements in HOMA‐IR and better fasting glucose control. These changes, detectable through breathomics, provide a noninvasive window into the impact of supplement use on metabolic health and glucose regulation in individuals with diabetes [88–91].

Breathomics platforms detect metabolic VOC signatures such as acetone (glycemic control), ethane and pentane (oxidative stress), and exhaled NO (vascular health), with reported predictive accuracies of 82%–85% for glucose variability and oxidative markers. VOC profiles dynamically reflect changes induced by supplementation and lifestyle interventions, offering real‐time feedback on metabolic improvements before changes appear in traditional blood tests. Integrating VOC technology with wearable and mobile health devices enables continuous, personalized diabetes management, enhancing adherence and outcome tracking.

## 5. Technological Advances

**Figure 5:**
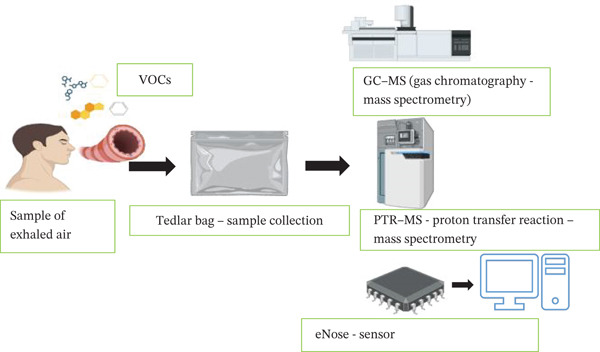
Schematic representation of breath VOC analysis in diabetes using GC‐MS, PTR‐MS, SIFT‐MS, and electronic nose (eNose) technologies. Breathed air is collected in a sample bag and analyzed using advanced analytical platforms or sensor‐based systems for noninvasive detection of key biomarkers.

Traditional methods for diagnosing and monitoring blood sugar and ketone levels in clinical settings rely on finger‐prick blood tests using test strips and electronic readers. These methods are invasive, painful, costly, and potentially unsafe if not handled properly. In contrast, breath analysis in humans provides a quick, noninvasive means of identifying organic volatiles associated with various disorders. Diabetes mellitus causes increased production of ketone bodies, including acetoacetate, *β*‐hydroxybutyrate, and acetone, during fat metabolism when glucose levels are low. Acetone, a key biomarker, is expelled through breath. Advanced techniques, such as GC‐MS, laser photoacoustic spectrometry, PTR‐MS, and SIFT‐MS, can be utilized for breath assessment (Figure 5, Table 4) [17, 96].

**Table 4:** Comparison of analytical techniques for breath analysis in diabetes monitoring.

Features	Principle	Advantages	Limitations	Portability	Sensitivity	Real‐time monitoring	Use case	VOCs identified	Clinical applications	Cost	Ref.
GC‐MS	Separates VOCs chromatographically; ions identified by mass spectrometry, including acetone, isoprene, ethanol, and aldehydes (sensitivity ppb‐ppt)	High sensitivity, comprehensive identification, and quantification; gold standard for validation and discovery	Bulky, nonportable lab instrument, expensive, requires trained operators, and slow due to sample prep & analysis time	Nonportable and laboratory‐based	High sensitivity	No (requires sample preparation and separation time)	Detailed VOC profiling; metabolomic biomarker discovery; VOC mixture characterization	Acetone (~0.1–20 ppm), isoprene (0.05–0.2 ppm), ethanol, aldehydes (e.g., hexanal), and isopropanol	Biomarker validation, metabolic phenotyping, ketoacidosis, and glycemic control research	Expensive	[13, 92]
PTR‐MS	Proton transfer reaction ionizes VOCs; ions detected by MS, identifies major diabetic VOCs like acetone and isoprene in real time (ppt sensitivity)	Real‐time detection, very high sensitivity, minimal sample prep, and suitable for dynamic metabolic studies	Bulky, high cost, limited isomer discrimination, and requires a skilled technician	Medium portability and lab environment–favored	High sensitivity, down to parts per trillion	Yes (real‐time monitoring possible)	Real‐time metabolic VOC monitoring; clinical research and exploratory diagnostics	Acetone, isoprene, ethanol, aldehydes, and other mass‐specific organic volatiles	Dynamic monitoring of metabolic changes and glucose/ketone monitoring	Expensive, bulky	[93, 94]
ENoses	Sensor arrays detect VOC mixtures’ pattern signatures analyzed by ML algorithms; detect acetone, isopropanol, and ethanol patterns with moderate specificity	Portable, rapid, cost‐effective, noninvasive, and suitable for point‐of‐care and continuous home monitoring	Lower chemical specificity and sensitivity, environmental interference, and requires calibration & advanced algorithms	Highly portable, wearable, and consumer device integration emerging	Moderate sensitivity	Yes (immediate pattern‐based reporting)	Screening, daily home monitoring, and early detection of hypoglycemia and ketoacidosis	Acetone, isopropanol, ethanol, and breath pattern recognition (complex VOC mixtures)	Noninvasive daily glycemic monitoring, hypoglycemia, and ketoacidosis early warning systems	Cost‐effective	[95]

### 5.1. GC‐MS

GC‐MS is a highly efficient analytical method, combining gas chromatography’s separation capabilities with mass spectrometry’s molecular identification. Various applications of GC‐MS include analyzing complex mixtures by providing detailed molecular profiles, including molecular weights, elemental composition, and structural properties. In breath acetone detection, it can become very time‐consuming because it requires lengthy sample preparation. For example, preparing acetone standards involves derivatization with a SPME needle, and each step takes approximately 20 min because acetone is a low molecular weight, highly volatile compound. Furthermore, breath samples must be collected in expensive Tedlar bags that can only preserve samples for 6 h or less. GC‐MS also has limited temporal resolution, as it takes several minutes to separate the components of a gas sample. In the case of breath acetone, the instrument also requires skill to operate, and training can be a barrier to its routine use for real‐time self‐monitoring in diabetes care [92]. GC‐MS has some advantages for breath analysis in diabetes research. It is a noninvasive, painless method that offers a pleasant alternative to blood tests. GC‐MS also offers rapid analysis times, enabling quicker diagnoses and real‐time monitoring. It has high sensitivity, which allows the detection of even small changes in VOC concentrations that tend to mirror metabolically linked changes associated with diabetes [97]. However, GC‐MS also has limitations. The equipment is costly and complex to operate, requiring extensive training in its use and in interpreting the data. Obtaining reliable data or results from the instrument requires careful sample handling and preconcentration protocols due to its limitations. The interindividual variation found in VOC profiles presents an additional barrier to interpreting VOC data, making it essential to ensure careful standardization for the isolation of interindividual samples [17, 98, 99].

### 5.2. PTR‐MS

PTR‐MS detects organic volatiles (VOCs) in air. This technology works by generating small protonated ions from many organic compounds (specifically, hydrogen or H_3_O^+^ in a flow‐drift tube under controlled and regulated conditions). The ionized molecules identified from the mass spectrometer reveal their mass‐to‐charge ratios. The great sensitivity of PTR‐MS, which detects VOCs down to parts per trillion, and the fact that it does not require sample preconcentration to measure (a significant advantage over time‐consuming techniques, e.g., GC‐MS) contribute to its ease of use, thereby eliminating ambiguity and lengthy time delays during shutdowns. As a real‐time, continuous online reading technology, PTR‐MS is advantageous for assessing VOCs over the long term; however, PTR‐MS has some limitations. It cannot distinguish between molecules with the exact same molecular weight; every substance with the same *m*/*z* value must be considered as a potential source of the signal. Furthermore, the apparatus is enormous and costly and requires the use of qualified personnel. These constraints limit its usefulness for diabetes patients’ daily, real‐time self‐monitoring, particularly in nonclinical or low‐resource settings [93, 94].

### 5.3. SIFT‐MS

Real‐time analysis of trace gases and exhaled breath is performed using SIFT‐MS. Breath specimens are ionized in a helium carrier gas by reactions with precursor H_3_O^+^, NO^+^, and O_2_^+^ ions. Chemicals can be measured at ppb concentrations without sampling or calibration by performing ion–molecule reactions over a specified time period and identifying the resulting ions by mass spectrometry. SIFT‐MS provides swift and precise breath analysis; however, it cannot differentiate between chemicals in complex gas mixtures. Furthermore, the equipment is nonportable, requires skilled staff, and is not suitable for elderly or illiterate patients who require simple, self‐operated diabetic monitoring devices [13].

### 5.4. eNoses

An eNose device detects specific organic volatiles present in exhaled air, including acetone, which is elevated in uncontrolled diabetes, offering a potential noninvasive method for identifying and tracking the condition. If diabetes is not adequately controlled, the body’s metabolism will revert to using fat instead of glucose. The metabolic pathway of fat yields acetone, which is further metabolized and expelled into the blood as waste, and can be detected in the breath. The eNose utilizes a biomarker‐sensitive sensor array and powerful pattern‐recognition algorithms to detect and quantify target substances in relation to biomarkers.

Several advantages of this technology include ease of real‐time monitoring, advances in early detection, and cost‐effectiveness compared to traditional diagnostics, such as blood tests. Using eNose technology can help recognize prediabetic status and support early interventions. eNose technology is minimally invasive and provides another convenient method of continuous surveillance to avoid repeated painful blood draws. Nevertheless, eNose systems still face significant challenges in providing accurate and reliable readings. Sensor drift, the gradual decline in performance over time, and variability in breath composition across individuals and under different conditions affect the data interpretation process. Additionally, confounding factors such as diet, smoking, or medication could also influence breath biomarkers and, therefore, negatively influence diagnosis. Although challenges lie ahead, eNose technology offers tremendous value for diabetes care. With ongoing advancements in sensor technology and the development of more precise pattern‐recognition algorithms, eNose systems will achieve greater accuracy. In the future, eNoses may be a critical component of large‐scale diabetes screening, early diagnosis, continuous monitoring, and even personalized management strategies, potentially significantly transforming healthcare.

eNoses utilize chemical sensors to detect organic volatiles in exhaled breath, mimicking the human sense of smell. The sensor responses are analyzed using pattern recognition algorithms to identify and quantify the chemicals present. eNoses have many benefits, including being nonintrusive and easy to use. They provide real‐time monitoring, are portable and inexpensive (compared to other methods), and require effortless sample preparation, so they can be taken anywhere or even be used at home. eNoses have the ability to detect multiple gases simultaneously.

However, eNoses also have limitations. They may not be as sensitive or specific as more sophisticated methods, such as GC‐MS and PTR‐MS. The environment can influence eNoses, requiring calibration at regular intervals to ensure measurement accuracy. Finally, eNoses may struggle to detect very low concentrations of certain gases in complex mixtures and to differentiate between similar substances closely related in molecular structure [95].

eNose technology uses an array of gas sensors and AI‐based pattern recognition models to detect and classify volatile compounds with increasing accuracy and robustness. Recent advances in machine learning, particularly neural networks such as convolutional neural networks (CNNs), deep neural networks (DNNs), and long short‐term memory (LSTM) networks, have significantly enhanced eNose capabilities for odor identification, both qualitatively and quantitatively. Feature extraction of the raw sensor signal, whether manual or learned from neural network representations, removes noise and redundancy to facilitate predictive quality models. For example, 1D‐CNNs that process the eNose signal in time series form achieve a 10% increase in classification accuracy compared to traditional classifiers such as support vector machines (SVMs) and random forests, as shown in the odor classification example. Other approaches treat sensor output as images for CNN processing, also achieving superior gas discrimination. The key challenges addressed by AI models include sensor drift over time and fluctuations in the environmental conditions. Drift compensation through adversarial training frameworks and feature selection algorithms enhances stability and robustness across different conditions and time points. Techniques such as discrete binary particle swarm optimization (DBPSO) help identify drift‐insensitive feature subsets, while deep belief networks assist in preprocessing to mitigate drift. Domain adaptation methods also enable models trained on one dataset to generalize well to others, a critical aspect for practical deployments.

The future prospects of eNose technology will thrive through integration with sophisticated AI algorithms that enable real‐time, accurate detection of complex odor mixtures. With increased availability and standardization of data, even deeper neural architectures may be able to improve predictive accuracy. In addition, applying drift compensation and domain adaptation procedures will further improve reliability and consistency over time. Owing to their rapid, noninvasive nature, eNoses are expected to become essential tools in medical diagnostics, environmental monitoring, and food quality assessment. Furthermore, combining eNoses with complementary sensing modalities and data fusion techniques will significantly expand their application potential [97, 100].

The regulatory framework for breath VOC analysis devices, including GC‐MS, PTR‐MS, and eNose systems, focuses on ensuring that the diagnostics are reliable, safe for patient use, and effective in the clinical context. In the United States, these devices are regulated by the FDA, with the pathway depending on intended use. Devices using labeled substrates, such as (^13^C)‐glucose, generally require premarket approval (PMA) due to the higher risk, while systems analyzing endogenous VOCs through integrated hardware typically follow the 510(k) clearance route by demonstrating substantial equivalence to approved devices. In Europe, CE marking under the medical device regulation (MDR) certifies conformity with health and safety standards, supported by clinical performance data and risk management. Clinically, these regulations support the safe adoption of breath VOC analysis in diagnostics. GC‐MS remains the gold standard for detailed metabolic profiling but is limited by cost and reliance on specialized laboratories, whereas PTR‐MS enables sensitive, real‐time monitoring. Emerging AI‐driven eNose systems provide promising noninvasive, point‐of‐care tools for metabolic monitoring, including diabetes detection and glycemic control. However, broader clinical translation requires standardized sampling protocols, large‐scale validation, environmental calibration, and harmonized regulatory guidance balancing innovation with patient safety [101].

## 6. Clinical Applications

**Figure 6:**
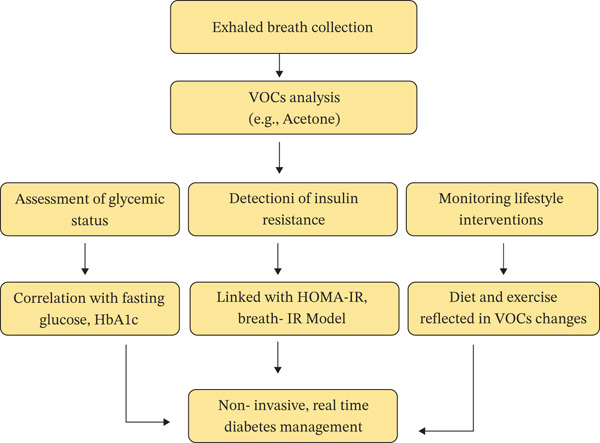
Workflow illustrating the role of exhaled breath VOC analysis in diabetes management: VOCs such as acetone are analyzed from exhaled breath to assess glycemic status, detect insulin resistance, and monitor lifestyle interventions, enabling noninvasive, real‐time diabetes management.

Profiling of breath biomarkers has proven to be a feasible, nonintrusive method for evaluating glycemic status and IR and monitoring the effectiveness of lifestyle changes. This nonintrusive analysis of breath provides information about metabolic mechanisms by measuring the organic volatiles in breath. These methods hold promise for early diagnosis and tailored management of diabetes and its associated complications [66]. The potential of breath biomarker profiling lies in its ability to reflect glycemic status, identify IR, and measure the effects of lifestyle changes. Research has shown strong associations between specific breath metabolites and glycemic status or IR. Noninvasive breath analysis technologies are available, facilitating continuous tracking of metabolic abnormalities and enabling more targeted therapy (Figure 6) [67].

### 6.1. Breath Biomarkers and Glycemic Status

Exhaled VOCs strongly correlate with clinically accepted glycemic metrics, such as fasting blood sugar, insulin, and HbA1c (an indicator of long‐term glycemic control), suggesting that breath biomarkers could be a valid, noninvasive method for measuring these metrics in place of blood tests. By utilizing ReCIVA technology, researchers published a study showing a “breath‐IR model” with 10 metabolites associated with HOMA‐IR, demonstrating that breath‐based metabolite profiling can reflect insulin sensitivity and glucose regulation. For example, a study using the ReCIVA breath sampling device identified a “breath‐IR signature” of 10 metabolites strongly correlated with HOMA‐IR (*R* = 0.95, *p* < 0.001) and the blood glycemic profile of fasting insulin (*R* = 0.91, *p* < 0.00) and fasting glucose (*R* = 0.80, *p* < 0.001), achieving an AUC‐ROC of 0.87, an accuracy of sensitivity 73%, and a specificity of 81%. This classification accuracy was comparable to benchmarks for HbA1c, providing further evidence for the potential of VOCs as usable, noninvasive markers for early detection and monitoring of IR and diabetes risk. Dietary interventions also alter VOCs, including reduced acetone and elevated isoprene levels, and glycemic and metabolic profiles. So integrating breathomics with digital glucose monitoring could enable real‐time, personalized tracking of metabolic health, though standardization and large‐scale validation remain essential for clinical adoption [67].

There are many potential clinical implications of these breath models, including early detection of IR, which could lead to early intervention before T2D develops. They could also provide a simple method to track disease progression and evaluate the effectiveness of a treatment regimen or change in lifestyle. They are easy to collect, enable rapid analysis, and may be suitable for large‐scale population health studies, thereby contributing to personalized and cost‐effective diabetes management [102, 103].

### 6.2. Breath Biomarkers and IR

Exhaled VOCs, including limonene, undecane, and 2,7‐dimethyl‐undecane, have been linked to IR. These metabolites correspond with the homeostatic model assessment of insulin, and breath analysis accurately reflects metabolic dysregulation. The breath‐IR model, which includes these VOCs, has been shown to differentiate between individuals with and without IR, suggesting potential as a noninvasive biomarker for IR, and could be helpful for early diagnosis, monitoring, and treatment of IR [104].

### 6.3. Breath Biomarkers and Response to Lifestyle Interventions

Breath tracking could be an innovative, nonintrusive tool for evaluating lifestyle changes to improve IR and glycemic control. Studies show that dietary changes and exercise have led to quantifiable changes in breath metabolite profiles, consistent with physiological changes indicating overall improvement in metabolic health. Breath metabolite changes can provide early information about a person’s biological response and could offer healthcare providers an opportunity to develop more individualized treatment plans moving forward. The ability to continuously monitor breath biomarkers enables real‐time analysis and optimization of outcomes in the management of diabetes and metabolic syndrome [103].

Breath biomarker profiles offer an attractive, noninvasive method for assessing glycemic status, evaluating IR, and monitoring patient responses to lifestyle interventions. Numerous breath metabolites (limonene and undecane) have been shown to correlate strongly with fasting glucose and HbA1c measurements, supporting the idea that breath‐based screening would provide a very rapid assessment of IR‐like characteristics for screening and monitoring. Breath biomarkers also indicate improvements in insulin sensitivity in response to interventions such as diet and exercise, providing an opportunity to plan longitudinal changes in real time based on breath. Nonetheless, breath sampling used in clinical practice requires further advancements in the reliability of X‐ray and portable analysis devices, standardized sampling devices, and protocols that adequately account for individual variation. In addition to these concerns, breath‐based testing has very promising potential to improve diabetes prevention and metabolic health management [105].

## 7. Challenges

The field of breathomics investigates the organic volatiles in breath but faces challenges in standardization, integration with personalized medicine, and predictive modeling. While consistent outcomes require standardization, personalized medicine demands consideration of individual heterogeneity in VOC profiles. Despite its potential for rapid, easy, and nonintrusive health assessments, further investigation is necessary to validate breathomics and integrate it with existing screening technologies [106].

Accurate profiling of organic volatiles relies heavily on standardized breath sample collection and analysis, as factors such as breathing patterns, collection timing, and environmental influences can substantially affect VOC composition. While online (real‐time) and offline (stored) sampling techniques exist, often using Tedlar bags, GC‐MS is the preferred VOC detection technique due to its excellent sensitivity and separation capabilities. However, GC‐MS involves complex sample preparation, requires expert handling, and is time‐consuming, driving ongoing efforts to standardize sampling and analytical procedures to improve reproducibility and comparability across clinical and research environments [107].

The significant individual differences in organic volatile profiles, influenced by genetics, lifestyle, and environment, make it difficult to identify universal biomarkers for diseases [108, 109]. The study of breathomics also faces several critical challenges. Initially, evaluating and interpreting organic volatile profiles is inherently complex, requiring advanced multivariate statistical methods to identify clinically meaningful patterns. Furthermore, the clinical applicability of breathomics is still limited. Extensive validation through large‐scale, multicenter trials involving diverse patient populations and disease states is necessary. Additionally, successfully integrating breathomics into current diagnostic workflows requires technological compatibility, data standardization, and real‐time interoperability.

Ethical concerns in breath‐based patient monitoring include issues related to data privacy and misuse, as the profiles of exhaled VOCs may expose sensitive health information beyond diabetes. Informed consent is essential for clarifying how biological data will be acquired, used, and stored. There are also current gaps in the standardization of breath sampling and analysis, which could lead to misinterpretation or diagnostic errors. Furthermore, AI‐based prediction models must be transparent and unbiased to avoid inequitable healthcare outcomes. Finally, regulatory oversight is needed to ensure breathomics technologies meet ethical, clinical, and data security standards before widespread adoption [110, 111].

## 8. Future Directions

• Standardization efforts

Establishing standardized protocols for breath VOC collection, analysis, and data reporting remains essential to ensure reproducibility and cross‐study comparability. Recent advances propose multistage workflows for VOC identification tailored to diabetes‐related compounds, enabling robust biomarker discovery and validation.

• Personalized medicine and predictive modeling

Personalized medicine is advancing through the integration of individual VOC profiles with clinical and genomic data, facilitating metabolic phenotyping and precision in diabetes management. AI‐powered predictive models analyzing complex VOC signatures have shown promise in early diabetes risk stratification and therapeutic response prediction, supporting increasingly tailored intervention strategies.

The development of AI‐powered algorithms to predict disease risk or progression based on patterns in exhaled organic volatile substances. Machine learning algorithms, including random forests and DNNs, are being developed to detect subtle shifts in VOC patterns that occur before the clinical onset of IR or T2D. For example, predictive models based on breath‐IR signatures have achieved over 80% accuracy in distinguishing individuals with IR, highlighting their potential as noninvasive screening tools.

• Multidisciplinary collaboration and technological advancements

Technological innovations, including high‐throughput GC‐MS methods and miniaturized, portable sensor arrays targeting key VOCs such as acetone, isopropanol, and limonene, are bringing breath VOC analysis closer to point‐of‐care and at‐home monitoring applications. Multidisciplinary collaboration across analytical chemistry, clinical endocrinology, engineering, and data science is vital for overcoming challenges in sensor specificity, environmental confounding, and biological variability.

• Training healthcare

Healthcare professional training programs are crucial for accurately interpreting VOC data and advancing breathomics findings to clinical relevance. For example, clinicians could use VOC‐based readouts to complement HbA1c or fasting glucose tests, enabling them to intervene with a patient sooner should they identify an abnormal metabolic shift in a sampled breath.

## 9. Conclusion

Breathomics is ideal for personalized medicine because it generates highly individualized data across numerous metabolic pathways. Breath biomarker research represents a promising, nonintrusive platform for revolutionizing diabetic therapeutic care. Driven by the urgent global need for rapid diagnosis, continuous monitoring, and real‐time feedback, breath analysis, particularly of VOCs, offers a compelling alternative to blood‐based testing for assessing glycemic status and IR. Demonstrating sensitivity to nutritional consumption, physical exercise, and pharmaceutical therapy, breathomics emerges as a valuable tool for evaluating lifestyle interventions. Moreover, his review underscores the potential clinical role of VOCs as noninvasive containers of biomarkers of glycemic control, IR, and oxidative stress in diabetes. Unlike conventional blood‐based assays, VOC profiling captures real‐time metabolic fluctuations influenced by diet, physical activity, and therapeutic response, offering a dynamic approach to precision diabetes management. Utilization of advanced analytical methods, such as GC‐MS, SIFT‐MS, and potential AI‐driven predictive models, is further improving reliability, sensitivity, and clinical applicability, bringing breath analysis closer to routine diabetes care. To ensure clinical translation, upcoming initiatives should focus on validating specific VOC panels, such as acetone, isoprene, limonene, and undecane, across multiple diabetic and prediabetic populations to ensure diagnostic reliability and reproducibility. Combining breathomics with digital health platforms and AI‐based predictive models will enable real‐time monitoring of metabolic state and early identification of IR. Additionally, developing standardized clinical protocols and training healthcare professionals in VOC sampling, analysis, and interpretation is crucial for reliable implementation. Continued interdisciplinary collaboration and advancements in analytical and sensor technologies are vital to transition breathomics from laboratory research to routine clinical practice, ultimately enabling personalized, noninvasive, and dynamic management of diabetes.

**Nomenclature**
AGE: advanced glycation end product
CGM: continuous glucose monitor
DKA: diabetic ketoacidosis
eNoses: electronic noses
FBG: fasting blood glucose
GC‐MS: gas chromatography–mass spectrometry
GSIS: glucose‐stimulated insulin secretion
HbA1c: hemoglobin A1c
HBP: hexosamine biosynthetic pathway
HOMA‐IR: homeostatic model assessment of insulin resistance
IMGU: insulin‐mediated glucose uptake
IRS: insulin receptor substrate
OGTT: oral glucose tolerance test
PI3K: phosphatidylinositol 3‐kinase
PKC: Protein Kinase C
PPG: postprandial glucose
PTR‐MS: proton transfer reaction–mass spectrometry
ReCIVA: respiration collector for in vitro analysis
ROS: reactive oxygen species
SCFA: short‐chain fatty acid
SDK1: Sphingosine‐Dependent Protein Kinase 1
T1D: Type 1 diabetes
T2D: Type 2 diabetes
TCA: tricarboxylic acid
VOC: volatile organic compound

## Author Contributions

Nathiya Ranganathan, J. Geetha, and Anbarasu Krishnan: investigation, methodology, formal analysis, software, visualization, validation, and writing—original draft. A. S. Vickram, A. Saravanan, Jeganathan Chinnadurai, Yi‐Hsun Chen, and Wei‐Chung Chen: data curation, resources, and visualization. I‐Chen Wu, Vinoth Kumar Ponnusamy, and Selvakumar Palaniappan: conceptualization, investigation, resources, supervision, and writing—review and editing.

## Funding

No funding was received for this manuscript.

## Disclosure

We confirm that the manuscript being submitted is original, has not been previously communicated to your journal, and is not currently under consideration by any other journal. It will not be submitted elsewhere before a decision is made. All authors agreed with the submission and confirmed that all its elements are in compliance with the journal’s publishing ethics policy. The “Guide for Authors” of the *Journal of Diabetes Research* has been followed in preparing the manuscript.

## Conflicts of Interest

The authors declare no conflicts of interest.

## Data Availability

Data sharing is not applicable to this article as no datasets were generated or analyzed during the current study. The literature survey reference papers can be shared upon request.
